# Trpc1 as the Missing Link Between the Bmp and Ca^2+^ Signalling Pathways During Neural Specification in Amphibians

**DOI:** 10.1038/s41598-019-52556-0

**Published:** 2019-11-05

**Authors:** Isabelle Néant, Ho Chi Leung, Sarah E. Webb, Andrew L. Miller, Marc Moreau, Catherine Leclerc

**Affiliations:** 1 Centre de Biologie du Développement (CBD), Centre de Biologie Intégrative (CBI), Université de Toulouse, CNRS, UPS, F-31062 Toulouse, France; 2 Division of Life Science and State Key Laboratory of Molecular Neuroscience, The HKUST, Clear Water Bay, Hong Kong, PRC

**Keywords:** Cell biology, Developmental biology

## Abstract

In amphibians, the inhibition of bone morphogenetic protein (BMP) in the dorsal ectoderm has been proposed to be responsible for the first step of neural specification, called neural induction. We previously demonstrated that in *Xenopus laevis* embryos, the BMP signalling antagonist, noggin, triggers an influx of Ca^2+^ through voltage-dependent L-type Ca^2+^ channels (LTCCs), mainly via Ca_V_1.2, and we showed that this influx constitutes a necessary and sufficient signal for triggering the expression of neural genes. However, the mechanism linking the inhibition of BMP signalling with the activation of LTCCs remained unknown. Here, we demonstrate that the transient receptor potential canonical subfamily member 1, (Trpc1), is an intermediate between BMP receptor type II (BMPRII) and the Ca_V_1.2 channel. We show that noggin induces a physical interaction between BMPRII and Trpc1 channels. This interaction leads to the activation of Trpc1 channels and to an influx of cations, which depolarizes the plasma membrane up to a threshold sufficient to activate Cav1.2. Together, our results demonstrate for the first time that during neural induction, Ca^2+^ entry through the Ca_V_1.2 channel results from the noggin-induced interaction between Trpc1 and BMPRII.

## Introduction

In vertebrates, neural induction occurs during gastrulation and represents the initial event in the formation of the nervous system. The dorsal and ventral ectoderm cells give rise to neural and epidermal progenitors, respectively. This binary choice of fate requires complex mechanisms and the action of both positive effectors, such as fibroblast growth factors (FGFs), and negative supressors, such as bone morphogenetic proteins (BMPs)^1^. A key event that occurs during the induction of the naïve ectoderm into neuroectoderm is the inhibition of the BMP signalling pathway by antagonizing factors, secreted by the dorsal mesoderm, such as noggin, chordin, follistatin, Xnr3 and cerberus^2^. So far, most neural induction studies have focussed on the identification of the transcriptional regulators. However, the early mechanisms that occur at the level of the plasma membrane still need identification. We have previously demonstrated in *Xenopus laevis* that neural induction is associated with Ca^2+^ influx through L-type voltage dependent Ca^2+^ channels (LTCCs)^3^, and that the resulting increase in intracellular Ca^2+^ concentration ([Ca^2+^]_i_) is necessary and sufficient to control the expression of neural genes and therefore to drive the ectoderm cells toward a neural fate^4–7^. Following our studies with *X. laevis* embryos, Ca^2+^ has subsequently been shown to be involved during neural induction in other vertebrate embryos such as zebrafish^8–10^, and chick^11^, as well as in some invertebrate species such as the ascidian *Ciona intestinalis* embryos where multiple Ca^2+^ transients were observed during the development of the neural plate^12^. Indeed, the maintenance of *C. intestinalis* embryos in low [Ca^2+^] conditions during gastrula and neurula stages impaired the development of the anterior neural plate.

In *X. laevis*, Ca^2+^ signalling via the activation of LTCCs was the first directly-visualized event that was linked to neural induction^13,14^. However, the mechanism of LTCC activation during neural induction remained an open question. LTCCs belong to the large family of voltage-activated Ca^2+^ channels; they are composed of a pore forming Ca_v_ subunit (Ca_V_1.x), associated with regulatory subunits, and regulate the influx of Ca^2+^ into cells during membrane depolarization. LTCCs are high-voltage-activated channels characterized by a threshold of activation at a membrane potential positive to −20 mV^15^. We previously showed that when noggin antagonizes BMP in *X. laevis* embryos, it triggers membrane depolarization and we suggested that it likely acts indirectly on LTCCs^16^. We proposed that there might be an intermediate factor, which links the inhibition of BMP with the activation of LTCC.

We previously demonstrated the presence of *trpc1* transcripts, and showed that they are restricted to the ectoderm of early blastula (stage 8) and early gastrula (stage 10.5) stage *X. laevis* embryos^16^. Trpc1 belongs to the canonical transient receptor potential (Trpc) family, itself part of the large family of Trp channels which are permeable to both Ca^2+^ and Na^+^^17^. Influx of Ca^2+^ and Na^+^ ions through Trp channels contribute to the membrane depolarisation, which in turn leads to the activation of Ca_V_1.x^18–20^ and to changes in cytosolic [Ca^2+^]. Interestingly, proteomic studies indicate that Trpc1 can interact with the carboxy-terminal domain of BMP receptor type II (BMPRII)^21^. We therefore suggest that the BMP-LTCC intermediate factor might be Trpc1.

Until now, the mechanistic relationship between the noggin-mediated antagonism of BMP signalling and the noggin-induced increase in cytoplasmic Ca^2+^, which occurs during neural induction, remains unclear. Here, we describe a possible mechanism by which BMP antagonism, either in the whole *X. laevis* embryo or in isolated ectoderm can activate LTCCs, and we show that in the ectoderm, Ca_V_1.2 is the main component of LTCCs. Our studies demonstrate that the inhibition of BMP signalling by noggin triggers a channel activation cascade, and that the modification of the dynamic interaction between BMPRII and Trpc1 is a central component of this mechanism. We propose that this interaction promotes an initial influx of cations through Trpc1, which then depolarizes the membrane of ectoderm cells up to the threshold of Ca_V_1.2 channel activation. Our new results suggest that Trpc1 might be the missing link in the pathway between BMPRII inhibition and Ca_V_1.2 channel activation.

## Results

### Ca_V_1.2 channels are expressed in the dorsal ectoderm

**Figure 1:**
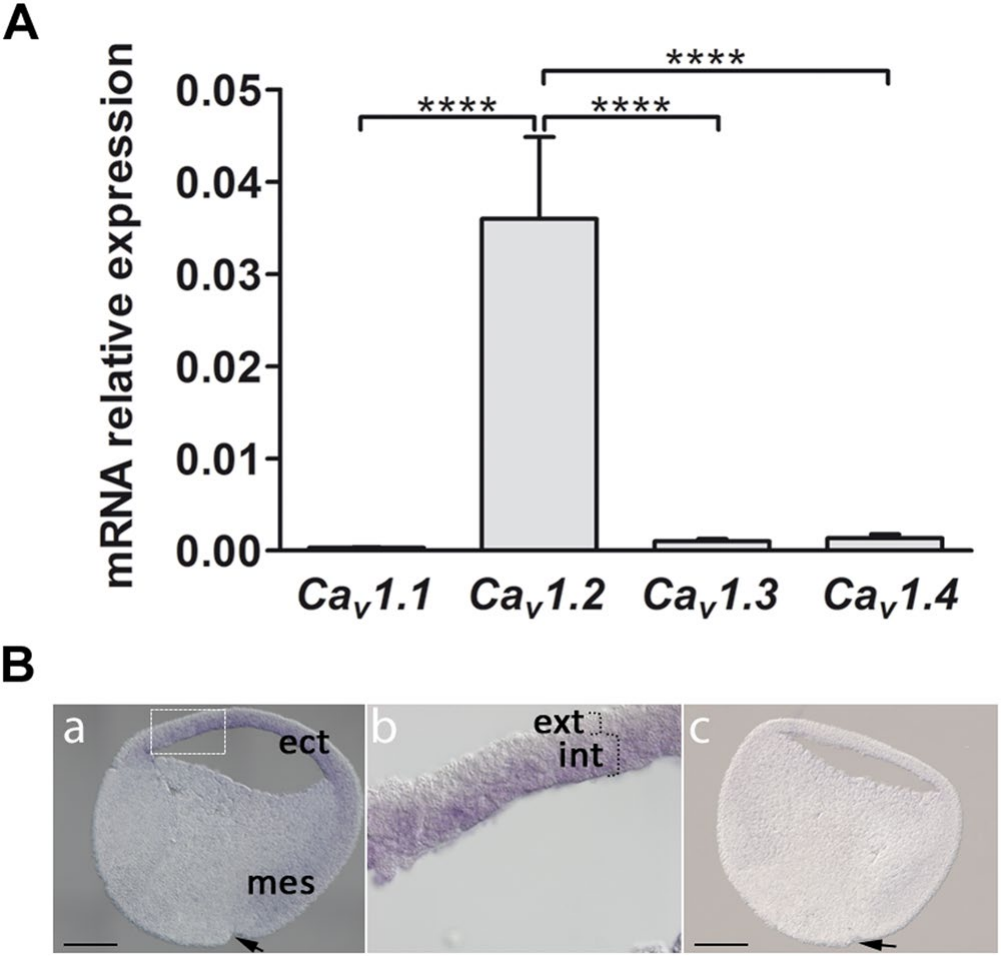
Expression of *Ca*_*V*_*1.2* mRNA in *X. laevis*. **(A)** RT-qPCR analysis of *Ca*_*v*_*1.1, Ca*_*v*_*1.2, Ca*_*v*_*1.3* and *Ca*_*v*_*1.4* in ectoderm (animal caps) isolated before gastrulation at stages 8 and 9. The expression levels were normalized to the housing keeping gene *odc (ornithine decarboxylase)*. The level *Ca*_*v*_*1.2* mRNA was significantly higher than that of *Ca*_*v*_*1.1*, *Ca*_*v*_*1.3* and *Ca*_*v*_*1.4* (one way ANOVA with Bonferroni’s test, ****P < 0.0001). The data represent mean ± SEM of 9 independent experiments such that 20 animal caps were used for each experiment. **(B)** *In situ* hybridization to show the pattern of localization of *Ca*_*V*_*1.2* mRNA in sections acquired at early gastrula (stage 10). **(Ba)** Photomicrograph of a sagittal section labelled with the anti-sense probe, which shows *Ca*_*V*_*1.2* expression in the ectoderm (ect) and dorsal mesoderm (mes). There is no *Ca*_*V*_*1.2* expression in the ventral mesoderm. **(Bb)** Higher magnification view of the ectoderm (corresponding to the white dashed rectangle in panel (**Ba**), which shows that *Ca*_*V*_*1.2* is expressed in the internal layer (int) but not in the external layer (ext). **(Bc)** Photomicrograph of a sagittal section showing no labelling with the sense probe. In **(Ba-Bc)**, dorsal is to the right and the arrows indicate the dorsal blastopore groove. Scale bars are 300 µm.

Previous direct visualization of the Ca^2+^ dynamics during neural induction in *X. laevis* embryos revealed the generation of spontaneous Ca^2+^ transients in the most anterior part of the dorsal ectoderm, and these were shown to be associated with the expression of functional LTCCs in the plasma membrane^7^. Here, using ectoderm isolated at the time of neural induction (animal cap), we analysed the expression of the four genes encoding the Ca_v_ subunit, namely *Ca*_*v*_*1.1*, *Ca*_*v*_*1.2*, *Ca*_*v*_*1.3* and *Ca*_*v*_*1.4*. As shown in Fig. 1A, the level of *Ca*_*v*_*1.2* mRNA is approximately 30 to 100 times higher than those of *Ca*_*v*_*1.1*, *Ca*_*v*_*1.3* and *Ca*_*v*_*1.4* (see also Supplementary Fig. S1A). This indicates that *Ca*_*v*_*1.2* is the major Ca_v_ subunit expressed in ectoderm isolated before gastrulation (i.e., at stage 8 and stage 9) or during gastrulation (i.e., at stage 10.5). In addition, no significant difference in Ca_v_1.2 expression was observed when comparing the mRNA levels in ectoderm isolated from stage 8, 9 or 10.5 embryos (Supplementary Fig. S1B). Furthermore, the expression of all four *Ca*_*v*_1 isoforms was not significantly altered in stage 8–9 animal caps following treatment with noggin (n = 11, Mann-Whitney test, see Supplementary Fig. S1C,D). To determine the spatial expression of *Ca*_*v*_*1.2*, we performed *in situ* hybridization (ISH) on sagittal sections of gastrula-stage embryos (stage 10), and showed that the mRNA was detected in the ectoderm and in the mesoderm. However, in the mesoderm it was restricted to the dorsal side of the embryo (Fig. 1Ba), such that no *Ca*_*v*_*1.2* expression was detected in the ventral mesoderm (Fig. 1Ba). Interestingly, within the ectoderm, the expression of *Ca*_*v*_*1.2* mRNA was restricted to the inner layer (Fig. 1Bb), which is the first layer to be induced toward a neural fate during gastrulation^22^.

These data demonstrate that the LTCCs implicated during neural induction are Ca_v_1.2 channels. Since these channels are voltage-operated Ca^2+^ channels (VOCCs), this brought our attention to identifying the mechanism by which Ca_v_1.2 channels are activated specifically in the dorsal ectoderm during the process of neural induction.

### Trpc1 is expressed in the ectoderm at the onset of neurogenesis

**Figure 2:**
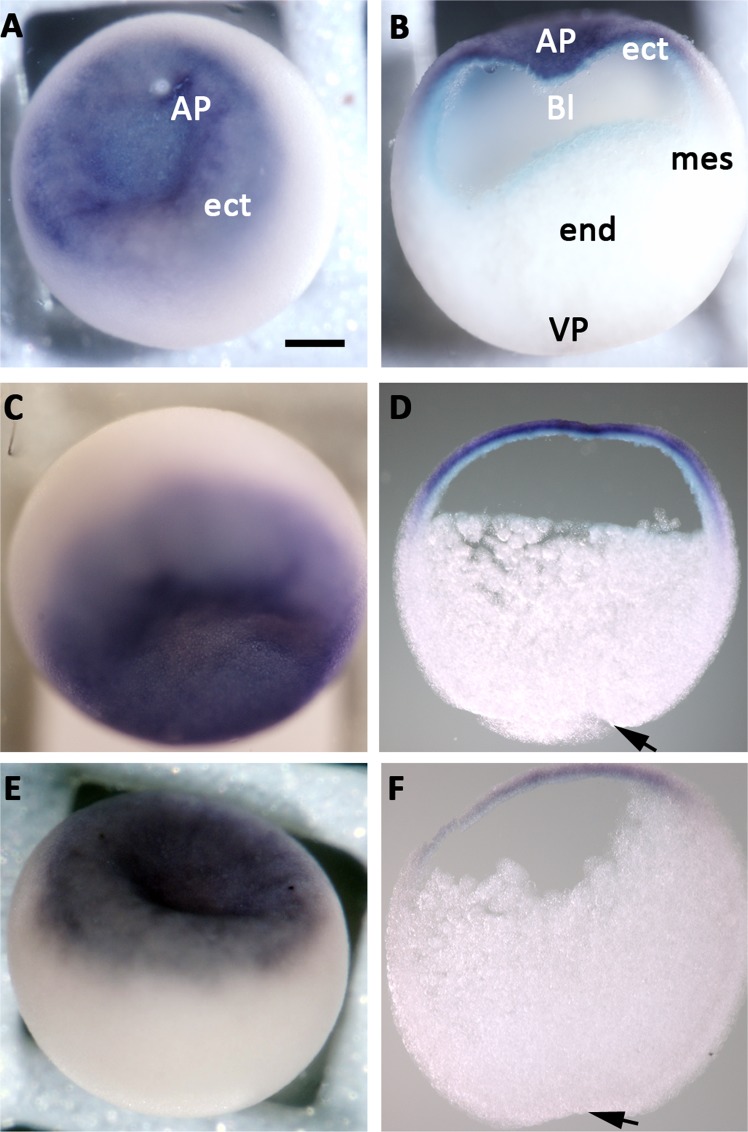
*trpc1* is expressed in the ectoderm of blastula and gastrula stage embryos. Spatial expression of *trpc1* mRNA in blastula and gastrula stage embryos. Whole mount *in situ* hybridization was performed on embryos fixed at blastula (stage 9), early gastrula (stage 10.5) and late gastrula (stage 11.5). **(A)** Expression of *trpc1* at stage 9 in an intact embryo (animal pole view), showing that the ectoderm is labelled. **(B)** Sagittal section taken through the embryo shown in panel A, indicating *trpc1* expression in the ectoderm alone; the mesoderm and endoderm were not labelled. **(C)** Expression of *trpc1* in an intact embryo at stage 10.5 (animal pole view). **(D)** Sagittal section taken through the embryo shown in panel **(C)**, showing *trpc1* expression in the ectoderm, with no difference in the level of expression between the dorsal and the ventral sides. **(E)** Expression of *trpc1* in an intact embryo at stage 11.5 (animal pole view). **(F)** Sagittal section taken through the embryo shown in panel E. AP, VP, ect, mes, end and Bl are animal pole, vegetal pole, ectoderm, mesoderm endoderm and blastocoel, respectively. In panels **(D)** and **(F)**, dorsal is to the right and the arrows indicate the blastopore lip. Scale bar is 300 µm.

**Figure 3:**
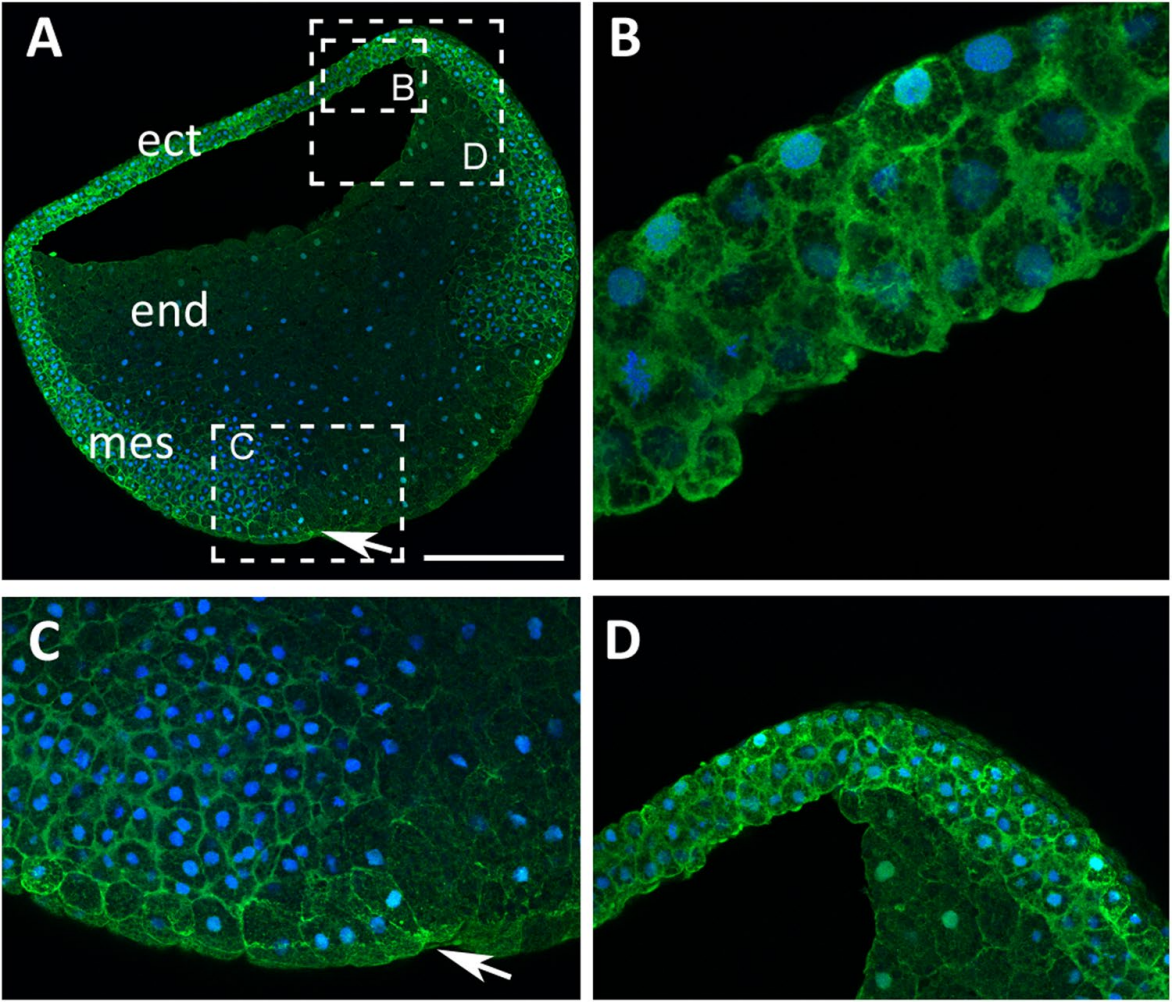
Pattern of localization of Trpc1 in gastrula stage embryos. **(A)** Single optical view of a sagittal section taken though a gastrula stage embryo, showing the localization of Trpc1 in the ectoderm (ect) and mesoderm (mes). There is a lower level of Trpc1 expression in the endoderm (end). The arrow indicates the position of the blastopore lip. The regions bounded by the dashed white rectangles are shown at higher magnification in panels (B–D). These images show that at the cellular level, Trpc1 is localized mainly in the plasma membrane. Trpc1 localization, shown in green, was revealed with the rabbit anti-Trpc1 polyclonal antibody and an Alexa-555-conjugated anti-rabbit secondary antibody. The nuclei (in blue) were labelled with ToPro3. Scale bars are 300 µm in panel (A), and 40 µm in panels (B–D).

We previously showed by RT-PCR analysis that *trpc1* transcripts are present in the oocyte, during the early blastula (stage 8) and early gastrula (stage 10.5) stage embryos, as well as in animal caps^16^. In this new study, we performed RT-qPCR analysis to determine if the expression of *trpc1* is modified at the onset of gastrulation and whether noggin regulates *trpc1*. No significant change in *trpc1* expression was observed in ectoderm isolated before gastrulation (stage 8 and 9) or at mid-gastrula (stage 10.5) (Supplementary Fig. S2A, n = 7). In addition, noggin did not affect the expression of *trpc1* (Supplementary Fig. S2B, n = 9). In order to obtain spatial information regarding the expression of *trpc1*, we performed whole-mount *in situ* hybridization and showed that during the blastula and gastrula stages, the expression of *trpc1* mRNA was restricted to the ectoderm and there was no difference between the dorsal and the ventral sides (Fig. 2A–F). To further demonstrate that this channel is expressed at the right time and place during neural induction, we performed immunohistochemistry on sagittal sections of late blastula/early gastrula stage embryos. We showed that Trpc1 is present in cells of the ectoderm (Fig. 3A,B,D) and the mesoderm (Fig. 3C), both in the cytoplasm and in the plasma membrane, whereas it is not detectable in the endoderm (Fig. 3A).

### Trpc1 regulates noggin-induced Ca^2+^ transients and membrane depolarization

**Figure 4:**
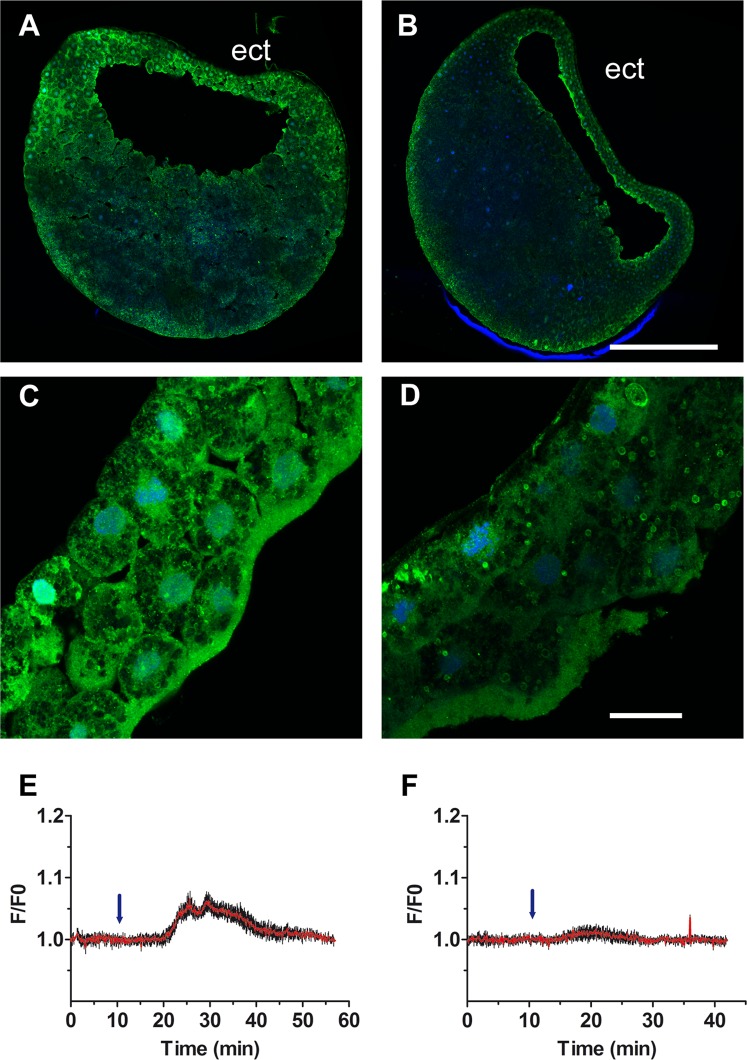
*Trpc1* knock down reduces the expression of Trpc1 in the ectoderm and abolishes the increase in intracellular Ca^2+^ generated following activation of Ca_V_1.2 channels in animal cap explants. **(A–D)** Embryos at the 2-cell stage were injected with **(A–C)** control-MO or **(B–D)** TRPC1-MO1 into both blastomeres, and the expression of Trpc1 was revealed by immunostaining at the blastula stage. **(A,B)** Confocal view of sagittal section taken through the entire embryo. (**C,D**) Confocal view at the level of the ectoderm. *trpc1* knock-down impaired the expression of Trpc1. The nuclei (in blue) were labelled with ToPro3. Scale bars are 350 µm in **(A,B)** and 40 µm in **(C,D)**. **(E,F)** Relative changes in fluorescence (F/F0) revealing changes in intracellular Ca^2+^ generated in single animal caps loaded with the Ca^2+^-indicator Fluo4 and isolated from embryos injected with either **(E)** control-MO or **(F)** TRPC1-MO1. The data are plotted as the mean of F/F0 (red traces) + SEM (black bars) from 7 **(E)** or 10 **(F)** randomly selected fields within a single animal cap. Noggin (2 µg/mL) was added (blue arrows) within the first 10 min after the start of data acquisition. Additional data are provided in Figure S3.

Evidence from the literature suggests that Trpc1 can form cationic channels by heteromultimerisation with other members of the Trp family such as Trpp2^23^ and Trpv4^24^, or with other transmembrane proteins such as Orai1 as part of store-operated Ca^2+^ entry complexes^25^. We therefore used RT-qPCR to determine the expression level of these partners in amphibian animal caps; *trpp2* and *trpv4* are indeed expressed at a comparable level to *trpc1* and *Ca*_*v*_*1.2*, whereas *orai1* expression is very low (Supplementary Fig. S2C). Because a long variant of Trpc1 also exhibits ion channel properties^26^, we performed a specific digestion assay and showed that in ectoderm cells it is only the long isoform that is expressed (Supplementary Figure S2D). To examine whether Trpc1 might play a role in the increase in intracellular Ca^2+^ observed during neural induction^7^, the expression of *trpc1* was inhibited using morpholino oligonucleotides (MO). Embryos at the 2-cell stage were injected into both blastomeres with either a standard control morpholino (CMO) or a translation blocking *trpc1* morpholino (TRPC1-MO1). Confocal microscopy revealed that TRPC1-MO1 inhibited the expression of *Trpc1* in the mesoderm and ectoderm (Fig. 4A–D). In addition, the Ca^2+^ increase in response to noggin was analysed in animal caps using the membrane permeant fluorescent Ca^2+^ probe Fluo4-AM. We previously showed that in animal caps, noggin triggers an influx of Ca^2+^ through LTCCs^3,5,27^. Here, we showed that the Ca^2+^ influx in response to noggin was completely abolished in animal caps isolated from TRPC1-MO1-injected embryos (n = 4; Fig. 4E,F and Supplementary Fig. S3). These data indicate that in addition to Ca_V_1.2, the noggin-induced Ca^2+^ influx requires functional Trpc1.

**Figure 5:**
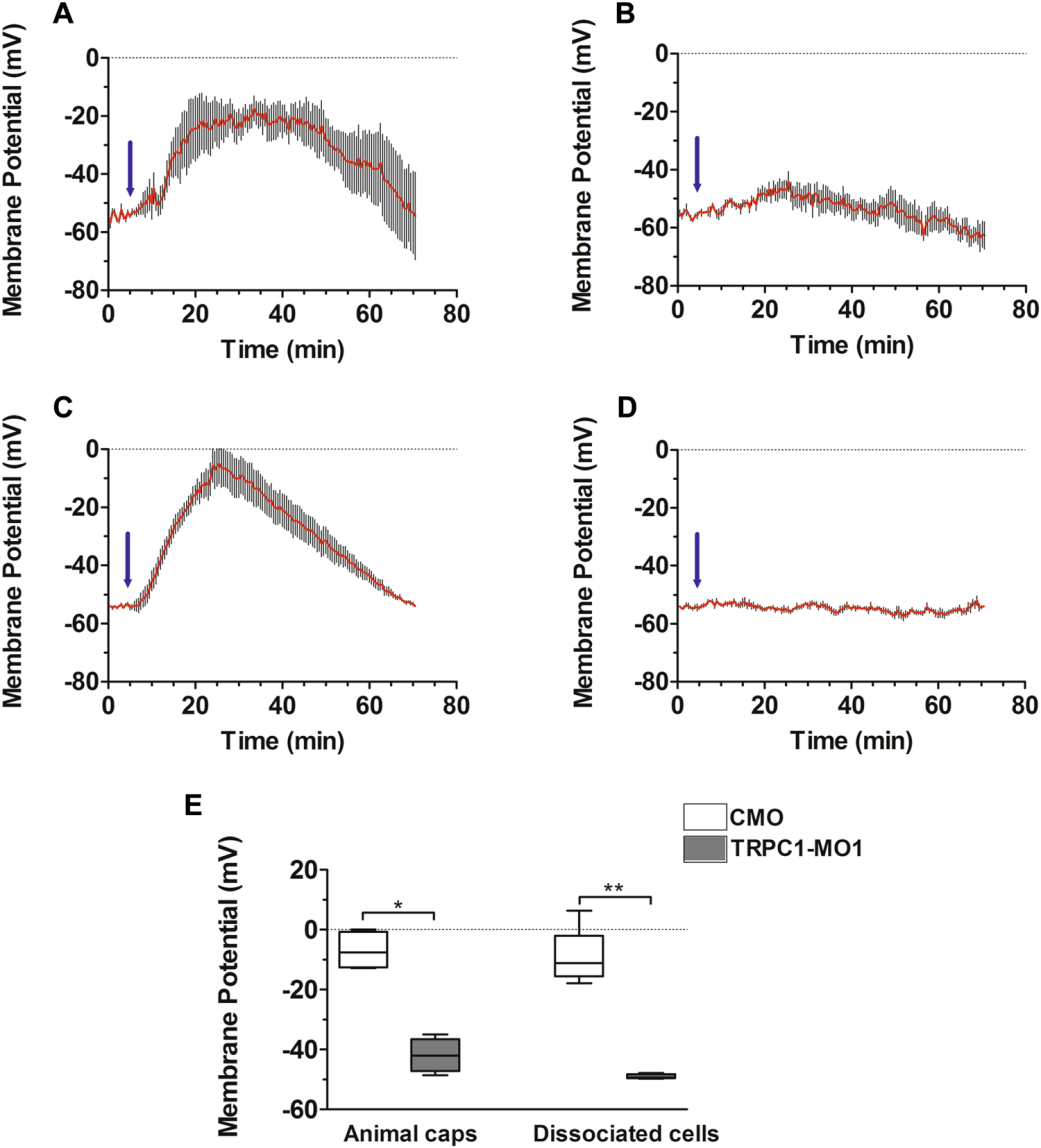
Membrane depolarisation induced by noggin in the ectoderm requires Trpc1. Control morpholino (CMO) or TRPC1-MO1 was injected into both blastomeres of 2-cell stage embryos. Animal caps were then prepared at late blastula (stage 9) and loaded with the potentiometric dye DiBAC4(3). **(A,B)** The mean calculated membrane potential revealed membrane depolarisation following the addition of noggin (3 µg/mL, see blue arrow) in animal caps prepared from **(A)** CMO- or **(B)** TRPC1-MO1-injected embryos (n = 4 for each). **(C,D)** The mean calculated membrane potential in isolated ectoderm cells (n = 5 cells) that were dissociated from animal caps prepared from **(C)** CMO- or **(D)** TRPC1-MO1-injected embryos. **(E)** A box plot showing the maximal depolarisation values reached after noggin stimulation of animal caps or dissociated ectoderm cells prepared from CMO-injected embryos (white bars) or TRPC1-MO1-injected embryos (grey bars). The maximal depolarisation values for the animal caps and dissociated cells prepared from CMO- and TRPC1-MO1 embryos were significantly different, (Mann-Whitney test with *P < 0.02 for animal cap recordings, and **P < 0.004 for dissociated cell recordings).

Since Ca_V_1.2 channels are VOCCs, we hypothesized that Trpc1 might contribute to the depolarization necessary to open these channels. Thus, animal caps were dissected at stage 9 and incubated with the potentiometric fluorescent dye DiBAC_4_(3), after which the fluorescence generated was visualized via confocal microscopy. Changes in fluorescence intensity were converted to changes in membrane potential in mV (described in detail in the Methods section). We showed that in animal caps prepared from CMO-injected embryos, noggin triggered membrane depolarisation from the resting calculated membrane potential of −54 mV to approximately −20 mV (Fig. 5A, n = 4 independent animal caps), a value sufficient to activate Ca_v_1.2 channels^15,28^. However, in animal caps prepared from TRPC1-MO1-injected embryos, noggin was unable to trigger a substantial change in membrane potential; at the most it reached −45 mV (Fig. 5B, n = 4 independent animal caps), which is not sufficient to activate Ca_v_1.2 channels^15^. To eliminate fluctuations due to movement of the animal cap cells, we also recorded the variation in membrane potential in cells dissociated from 15 animal caps dissected at stage 9 from CMO- or TRPC1-MO1-injected embryos. Similar to the data obtained from animal caps, after noggin stimulation the membrane potential depolarized to approximately −10 mV in the CMO-dissociated cells whereas no depolarization occurred in the TRPC1-MO1 dissociated cells (Fig. 5C,D, n = 6). As shown in Fig. 5E, the maximal depolarisation reached in the CMO animal caps and in CMO dissociated cells was −9.2 mV ± 3.9 mV (n = 4) and −10.9 mV ± 3.4 (n = 6), respectively. These values are in the range of the membrane potentials required to activate neuronal Ca_v_1.2 channels^28^. In contrast, in TRPC1-MO1 animal caps and dissociated cells, the maximum membrane depolarization recorded was −43.5 mV ± 2.6 mV (n = 4) and −50.4 ± 0.3 (n = 6), respectively, which are both well below the threshold voltage required to activate Ca_v_1.2.

Altogether, these results indicate that noggin can stimulate a depolarization of the membrane that reaches the activation threshold of Ca_v_1.2 channels and that this depolarization requires functional Trpc1. Our data therefore suggest that Trpc1 might be an intermediary between the action of noggin (i.e., BMP inhibition) and the activation of Ca_V_1.2 channels. This indicates a possible link between the BMP receptor (BMPR), Ca^2+^ transients and neural specification.

### Trpc1 regulates neural specification

**Figure 6:**
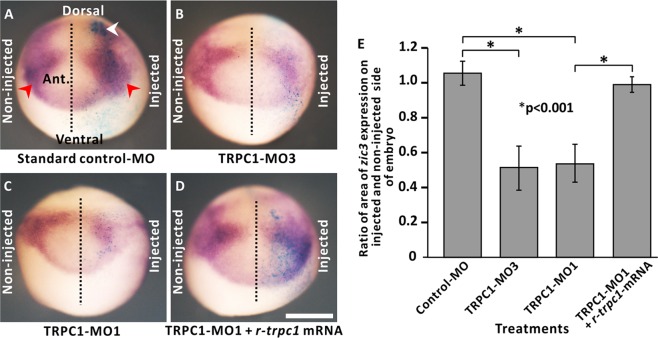
*trpc1* knock-down impairs the expression of the early neural gene, *zic3*. Embryos were co-injected at the 8-cell stage into a single dorsal animal blastomere with nuclear β-galactosidase mRNA and: **(A)** the standard control-MO, **(B)** a splice-blocking MO (TRPC1 -MO3), **(C)** TRPC1*-* MO1, or **(D)** TRPC1- MO1 plus *r-trpc1* mRNA. Embryos were then fixed at stage 14 for subsequent whole-mount *in situ* hybridization of *zic3;* see red arrowheads in panel **(A)**. The side of the embryo injected with MO ± *r-trpc1-*mRNA was confirmed by reaction of β-galactosidase with X-Gal, as shown by the blue labelling on the right side of each embryo; see white arrowhead in panel **(A)**. Scale bar is 500 µm. **(E)** A bar chart showing the mean ± S.E.M. (n = 17) ratio of the area of *zic3* expression on the injected and un-injected sides of the embryo. The asterisks indicate data that are significantly different at *p* < 0.001 when using one-way ANOVA and the Tukey’s honest significance post-hoc test.

To verify the proposed essential role played by Trpc1 in neural induction, we injected a single dorsal animal blastomere of embryos at the 8-cell stage with the CMO, the TRPC1-MO1, a splice-blocking morpholino (TRPC1*-*MO3), or TRPC1*-*MO1 plus a MO-resistant *trpc1* mRNA (*r-trpc1)*. The embryos were allowed to develop to the early neurula stage at which time the expression of *zic3*, the primary neural regulator characteristic of neural induction in *X. laevis*^27,29,30^, was determined. We showed that on the TRPC1-MO-injected side of the embryos (when using either TRPC1-MO1 or TRPC1*-*MO3), the expression of *zic3* was dramatically reduced when compared with the uninjected side (Fig. 6A–C). However, when TRPC1*-*MO1 was co-injected with *r-trpc1* mRNA, the expression of *zic3* was rescued (Fig. 6D,E). To confirm the effect of Trpc1 knock-down, we also examined the spatial expression pattern of *sox2* in embryos injected with either the CMO or the TRPC1-MO1. *Sox2* is a member of the SoxB1 subgroup of transcription factors; it is expressed in the dorsal ectoderm, is essential for neural development^31^ and its expression is controlled by Kcnip1, a Ca^2+^-dependent transcription factor^32^. The expression of *sox2* was markedly decreased in embryos injected with TRPC1-MO1 but not in those injected with CMO (Supplementary Fig. S4). This underscores the important role played by Trpc1 in neural induction.

### BMPRII and Trpc1 physically interact through the BMPRII-tail domain and the BMP antagonist noggin increases this interaction

**Figure 7:**
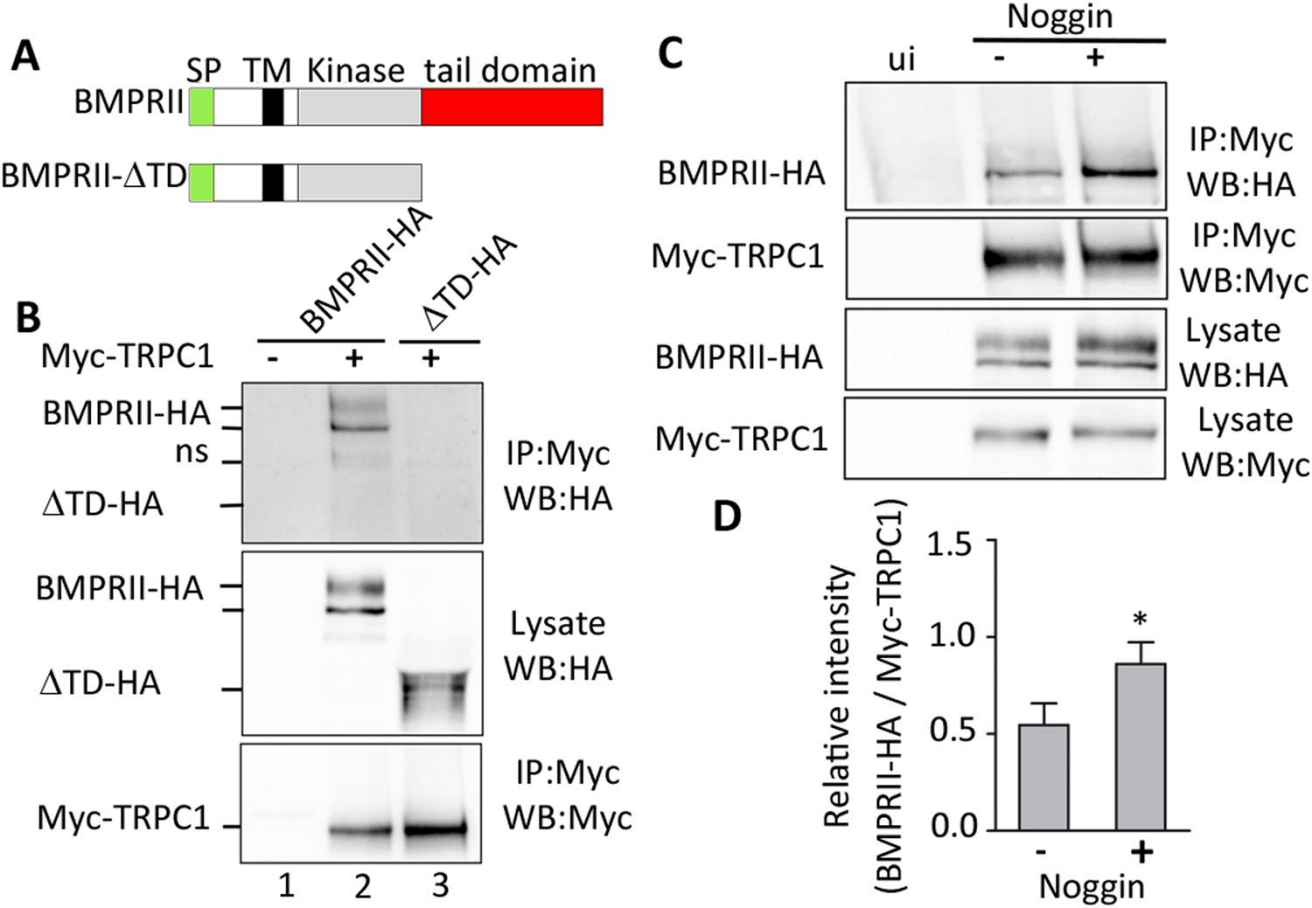
The tail domain of BMPRII is essential for the interaction with Trpc1, and noggin modulates the BMPRII-Trpc1 interaction. **(A)** Schematic illustration of the full-length BMPRII and the tail domain-deleted construct (BRII-∆TD). The yellow, black, grey and red rectangles show the signal peptide (SP); transmembrane domain (TM), kinase domain, and tail domain respectively. **(B)** Representative western blots showing the immunoprecipitation data acquired when analysing the protein-protein interaction between BMPRII and Trpc1. Embryos at the 4-cell stage were either injected (+) or not (−) with *BMPRII-HA* (200 pg/cell) or *BRII-∆TD-HA* (200 pg/cell), along with *Myc-Trpc1* (200 pg/cell) into all the blastomeres. Animal caps were then prepared at stage 8–9, lysed and subjected to immunoprecipitation. ns, non-specific band. Western blot data were revealed by enhanced chemiluminescence (ECL; n = 4 independent experiments). **(C**) Representative western blots showing the interaction between BMPRII and TRPC1 in the presence of noggin. Animal caps were collected at stage 8–9 from embryos injected with *BMPRII-HA* (200 pg/cell) and *Myc-Trpc1* (200 pg/cell) into all the blastomeres at the 4-cell stage, after which they were either incubated (+) or not (−) with 2 µg/mL noggin for 15 min, and then lysed and subjected to immunoprecipitation with anti-Myc antibody. The western blot data were revealed by ECL (n = 3 independent experiments). **(D)** Quantification of the BMPRII-HA fraction associated with Myc-Trpc1. Ratios of co-immunoprecipitated BMPRII-HA: Myc-Trpc1 were calculated in the absence or presence of noggin using the Bio-Rad ChemiDoc Image Lab software 5.2.1. There data represent the mean ± from 3 independent experiments. The asterisk indicates data that are significantly different at *p* < 0.05 when using the Mann-Whitney test. The full-length blots are presented in Supplementary figure S7.

Proteomic data suggest that Trpc1 physically interacts with BmprII at the tail domain^21^. We therefore examined the expression of BmprII and Trpc1 at the subcellular level in the ectoderm. Embryos at the 2-cell stage were injected with the HA-tagged-*bmprII* mRNA (*bmprII*-HA), after which immunohistochemistry was conducted in animal caps prepared from these embryos to show the expression of Trpc1. Our data showed that Trpc1 and BmprII-HA were both localized in the plasma membrane and that Trpc1 was also localized in the cytoplasm (Supplementary Fig. S5). To further analyse the relationship between Trpc1 and BmprII, we tested whether these proteins might form a complex in the ectoderm by performing a co-immunoprecipitation. Thus, all of the blastomeres in 4-cell stage embryos were co-injected with *myc-tagged trpc1* mRNA (*myc-trpc1*) and mRNA for the full-length *bmprII-HA* (Fig. 7A), and animal caps were dissected at stage 9. In this assay, BmprII-HA was efficiently precipitated with myc-Trpc1 (Fig. 7B, lane 2). In a murine system, glutathione-*S*-transferase pull-down assays indicate that the C-terminal tail of BmprII is a docking site for several proteins, including Trpc1^21^. Thus, in order to better characterize the physical interaction between BmprII and Trpc1 in our system, 4-cell stage embryos were co-injected with *myc-trpc1* mRNA and mRNA for a truncated form of BmprII, such that the C-terminal tail domain (TD) was deleted (*bmprII-ΔTD-HA*). Because a dominant-negative form of BmprII lacking the entire intracellular domain has been shown to have direct neuralizing activity^33^, we first tested whether the truncated form of BmprII used in this study, which lacked the C-terminal tail domain only (BmprII-ΔTD), had any neuralizing activity. Therefore, 2-cell stage embryos were injected with *bmprII-ΔTD* mRNA and animal caps were isolated at stage 9, after which we analysed the expression of *zic3*^30^ and *sox2*^34^, which are reported to be involved in the specification of neural fate, and of *bmp4*^35^ and *msx1*^36^, which control epidermal fate. In addition, *msx1* is an immediate early response gene to BMP4. Supplementary Fig. S6 shows that in animal caps over-expressing *bmprII-ΔTD*, the profiles of expression for *zic3*, *sox2* and *msx1* are significantly different from those of noggin-treated animal caps (n = 8 independent experiments, one way ANOVA with Bonferroni’s test, **P < 0.001, ****P < 0.0001). These data strongly suggest that in animal caps, BmprII-ΔTD is not able to convert ectoderm cells from an epidermal to a neural fate. Next, we used this truncated BMP receptor to test for the interaction between Trpc1 and the C-terminal tail domain. As shown in Fig. 7B (lane 3), the truncated form, BmprII-ΔTD-HA, was not precipitated with myc-Trpc1. This indicates that Trpc1 associates with BmprII in ectoderm cells, and that the C-terminal TD of BmprII contributes to the physical interaction between the two proteins.

Since noggin is able to trigger Ca^2+^ influx through Trpc1 channels, we investigated if it might be able to modify the interaction between Trpc1 and BmprII. Thus, animal caps from stage 9 embryos that had been co-injected with *myc-trpc1* mRNA and *bmprII-HA* mRNA were incubated in the presence or absence of noggin for 15 min prior to the co-immunoprecipitation assay. We showed that the presence of noggin significantly increased the co-immunoprecipitated fraction of BmprII by 1.64 fold (n = 3, p < 0.05; Fig. 7C,D).

Together, these results indicate that in the ectoderm, Trpc1 channels physically interact with the tail domain of BmprII, and this physical association is enhanced by noggin.

## Discussion

One of the first molecular mechanisms identified that regulates neural fate in the dorsal ectoderm of *X. laevis* embryos, was the inhibition of BMP signalling^37^ by molecules secreted by the dorsal mesoderm such as noggin, which are known to antagonize the activities of BMP2 and BMP4^38^. In previous studies, we showed that in addition to its physical interaction with BMP4, noggin triggers an increase in intracellular [Ca^2+^]_i_. via the activation of LTCCs^7,27,39^. However, the mechanism linking the inhibition of BMP signalling with the activation of LTCCs was unknown. Here we propose a mechanism explaining how the inhibition of BMP signalling by noggin might activate LTCCs.

This present study provides evidence that activated LTCCs are likely to be Ca_V_1.2 channels. Among the four *Ca*_*v*_*1.x* isoforms, only transcripts for Ca_v_1.2 are expressed at a high level and at the right place (i.e., in the inducible layer of the ectoderm and in the dorsal mesoderm), throughout neural induction. The activation of Ca_v_1.2 channels occurs after noggin binds to BMP4^40^; however, to date there is no evidence to suggest that noggin can directly activate Ca_V_1.2 channels. We therefore propose the existence of an intermediate factor between the BMP receptor and Ca_V_1.2 channels. Previously, we provided evidence showing that the Trp family member, Trpc1, is expressed in ectoderm cells during the blastula and gastrula stages^16^. Here, we analysed in further detail, the expression, localisation and function of Trpc1 during neural induction. We found that Trpc1 proteins are localized to the plasma membrane in the ectoderm and that the inhibition of *trpc1* expression by specific MOs abolished this distinct pattern of Trpc1 localization. Inhibition of *trpc1* also blocked the Ca^2+^ transients induced by noggin, and strongly reduced the expression of the early neural markers *zic3* in whole embryos. These data suggest that Trpc1, which is required for the conversion of the ectoderm into neuroectoderm, may form channels allowing Ca^2+^ influx. It is still not clear whether Trpc1 monomers can assemble to form functional channels. Studies performed in cell culture indicate that Trpc1 can form channels by interacting with other Trps or with Orai^41^. In particular, Trpc1 can assemble with Trpp2^23^ and Trpv4^24^. Here, we show that both Trpp2 and Trpv4 are expressed at high levels in the ectoderm before gastrulation, suggesting that Trpc1 might indeed form heteromeric channels with them. However, we have previously shown that Trpp2 only starts to be detected at stage 11 (mid-gastrulation), which is 5 hours after the stage we use the animal caps (i.e., stage 9)^42^. In addition, considering the low level of *orai1* expression, an interaction between Trpc1 and Orai1 in stage 9 ectoderm cells is unlikely. Although the formation of functional heteromeric Trpc1/Trpp2 channels is not excluded, our data clearly demonstrate that Trpc1, whatever its partner, is absolutely required for the increase in intracellular Ca^2+^ recorded in ectoderm cells following noggin stimulation, and suggest that Trpc1 is acting upstream of Ca_v_1.2 activation. Ca_v_1.2 channels are dihydropyridine (DHP)-sensitive channels, which can be directly activated by the DHP agonist (S)-BayK644^43^, and they are also classified as being high-voltage-activated channels. Neuronal Ca_v_1.2 channels have been reported to have activation midpoints (V_0.5_; i.e., the voltage required for half-maximal activation) of −17 mV^15^. Here, we show that in animal caps or dissociated ectoderm cells, noggin triggers depolarization of the membrane potential to around −10 mV, a value compatible with the published activation potential for Ca_V_1.2^15,28^. Furthermore, MOs directed against *trpc1* completely blocked the noggin-induced depolarization. Altogether these data clearly indicate that Trpc1 is a central node, which links the action of noggin to the activation of Ca_V_1.2 channels.

**Figure 8:**
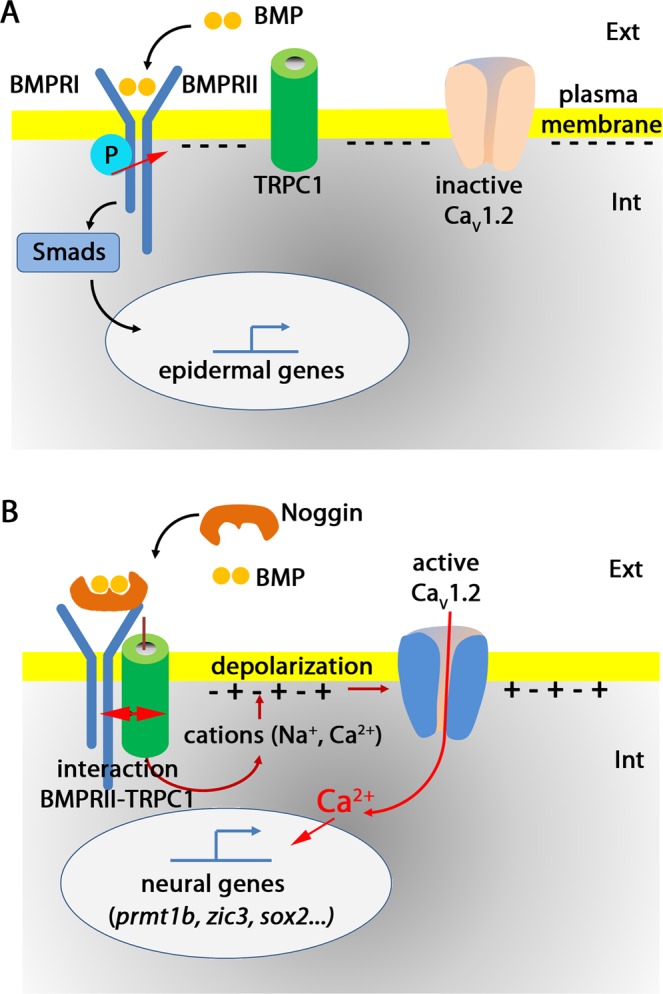
Hypothetical model to depict the role of Trpc1 channels in linking the inhibition of BMP pathway by noggin, and the activation of Ca_v_1.2 channels in ectodermal cells. During gastrulation, the cells of the embryonic ectoderm have the choice between two fates; they can give rise to either epidermal or neural progenitors. In the plasma membrane, the molecular components involved in this choice are BMP receptors type I (BmprI) and type II (BmprII), Trpc1 and voltage-dependent Ca^2+^ channels (Ca_v_1.2). The membrane potential in the ectoderm is ~−60 mV; i.e., the interior is negatively charged^62^. (**A**) Induction of the epidermis occurs through a signalling cascade, which involves the binding of Bmp4 to its receptor, and then the transphosphorylation of BmprII by BmprI. This is followed by the activation of Smads, which translocate into the nucleus to form active transcriptional complexes to control the expression of epidermal genes. In this scenario, there is no interaction between Trpc1 and BmprII, and Ca_v_1.2 remains inactive. (**B**) During neural induction, noggin binds to BMP4, and thus prevents the activation of the BMP pathway. As a consequence, it induces a physical interaction between BmprII and Trpc1 channels. This interaction leads to the activation of Trpc1, which either alone or associated with other Trp channels (e.g., Trpv4), triggers an influx of cations (Ca^2+^ and Na^+^). This influx of cations depolarizes the membrane (i.e., there is more positive charge inside) up to a threshold sufficient to open the voltage-gated Ca^2+^ channel, Ca_v_1.2. As we have previously shown^27,29^, the resulting influx of Ca^2+^ is then sufficient to activate the expression of downstream neural specific genes, such as *prmt1b*, *zic3* and *sox2*.

BMPs belong to the transforming growth factor-β (TGF-β) family and bind to type I and type II serine-threonine kinase receptors (i.e., BmprI and BmprII, respectively), both of which are required for signal transduction. After BMP binds to BmprI, the transduction of signal from the plasma membrane to the nucleus involves the transphosphorylation of BmprI by BmprII, and the activation of Smads^44^. This mechanism controls the epidermis fate in *X. laevis* ectoderm^35^ (Fig. 8A). It is generally acknowledged that the function of noggin is to inhibit BMP signalling by physically interacting with BMP2 and BMP4^40^. In previous studies, we provided evidence that noggin indeed has a dual function; (1) to inhibit BMP signalling, which leads to the repression of epidermal genes, and (2) to activate Ca^2+^ influx and thus elevate the intracellular [Ca^2+^]_i_ (Fig. 8B). We have previously demonstrated that this initial Ca^2+^ increase directly induces the expression of downstream neural specific genes, such *prmt1b* and *kcnip1*, which in turn activate other neural genes such as *zic3*, *sox2* and *p54nrb*^13,29,32,45^.

Here, we further analysed the mechanism by which noggin triggers the activation of Ca^2+^ signalling and established that, in naïve ectoderm cells, Trpc1 interacts with the C-terminal domain of BmprII. Furthermore, when neural induction was triggered by noggin, the interaction between BmprII and Trpc1 was increased by 1.6 fold. This enhanced interaction might then stimulate the activity of Trpc1. As a consequence, the membrane potential depolarizes to a threshold sufficient to open Ca_V_1.2 channels and the resulting increase in intracellular [Ca^2+^]_i_ activates the neural specific genes^29,32^ (Fig. 8B). Importantly, it should be noted that in *X. laevis*, Ca^2+^ is a permissive, necessary and sufficient signal to switch the ectoderm from an epidermal to a neural fate. We have shown that the inhibition of Ca_v_1.2 with a DHP antagonist prevents this conversion, and conversely, the direct activation of Ca_v_1.2 channels with the DHP agonist induces ectoderm cells to adopt a neural fate^39^

Evidence from the literature suggests that the effect of noggin on Ca^2+^ signalling is recapitulated in other cellular systems and that it also involves the interaction between Trpc1 and BmprII. For example, in pulmonary arterial smooth muscle cells, the inhibition of BMP by noggin results in the expression of Trpc1 and Trpc6, as well as an increase in the basal [Ca^2+^]^46^. Interestingly, in cultured embryonic *X. laevis* spinal neurons, BmprII and Trpc1 channels are both involved in an attraction-to-repulsion switching mechanism that occurs in response to a gradient of BMP7^47^.

In summary, the fact that an increase in [Ca^2+^]_i_ is a determinant in the process of neural induction has been shown in a number of different animal models^8,10–12^. However, to advance our understanding of the events that trigger neural induction, it was critical to elucidate the molecular mechanisms by which an antagonism of BMP signalling can elevate the [Ca^2+^]_i_. Here, we have identified a mechanism by which the inhibition of BMP signalling can regulate intracellular Ca^2+^ homeostasis and, as a result, subsequent gene expression (Fig. 8B). This mechanism therefore constitutes a possible missing link to explain how noggin might activate Ca^2+^ signalling during neural induction.

## Methods

### Ethical statement

In France, animal care and experimentation were conducted in accordance with protocols approved by The French “Ministère de l′Education Nationale, de l′Enseignement Supérieur et de la Recherche” and by the local Animal Care and Use Committee (authorization A31-555-01). Experiments conducted in Hong Kong were performed in accordance with the guidelines and regulations set out by the Animal Ethics Committee of the HKUST and by the Department of Health, Hong Kong.

### Embryos

*Xenopus laevis* embryos were obtained and staged using standard procedures^29,48^. Embryos were raised in 0.1X Normal Amphibian Medium (NAM)^49^. Presumptive ectoderms (animal caps) were dissected from blastula stage embryos (stage 8–9) lying on a cushion of 1% agarose and immersed in 0.5X NAM, using watchmaker’s fine forceps and a platinum microsurgery tip of 400 μm width (Xenotek Engineering, Belleville, IL, USA). All incubations were conducted at ~20 °C. Mouse recombinant noggin/Fc chimeric protein (at 50 μg/mL; R&D system) was prepared in PBS containing 0.1% BSA. Animal caps were treated with 2 μg/mL noggin/Fc in 0.5X NAM, as previously described^16^.

### Animal caps

Animal caps consist of ectodermal tissue that is isolated from the embryo at the blastula stage. At this stage, animal cap cells are multipotent and exhibit developmental plasticity. In the absence of an inducing signal the ectodermal cells express markers specific to epidermis. However, in the presence of BMP antagonists, such as noggin, they express neural-specific markers^50^. For some of the membrane potential measurements, we used the dissociated cells of animal caps. Animal cap cell dissociations were performed by a simple 15-min incubation in Ca^2+^, Mg^2+^ free 1× Steinberg’s solution (CMFSS: 58 mM NaCl, 0.67 mM KCl, 4.6 mM Tris-HCl at pH 7.4)^51^.

### Morpholino oligonucleotides and microinjections

Two morpholino oligonucleotides (MO; GeneTools, Corvalis, USA) were designed to block the translation (TRPC1-MO1) and splicing (TRPC1-MO3) of *X. laevis trpc1*. The TRPC1-MO1 sequence has previously been characterized for its specificity and efficacy in knocking down the endogenous *X. laevis* Trpc1 protein^52^. The TPRC1-MO3 sequence was chosen at the splicing site of exon 6 as follows: 5′-AGCACCTTCCCAGACTCCTACCTCC-3′. Standard control morpholino (CMO) was provided by the manufacturer (5′-ATGGAGGCAACTGTCGTCGCTACGA-3′). Standard CMO (17 ng), TRPC1-MO3 (10 ng), TRPC1-MO1 (17 ng) or TRPC1-MO1 (17 ng) plus *r-trpc1*-mRNA (200 pg), were pressure-injected into a single dorsal animal blastomere of embryos at the 8-cell stage. To identify the injected side, *EGFP* mRNA or *nlacZ* (150 pg) were used as tracers. The *lacZ* expression was revealed with the X-Gal or Red-gal substrates (Research Organics).

### *Trpc1* plasmid constructs and *in vitro* transcription

The wild type *X. laevis trpc1* open reading frame (ORF) was amplified by PCR from a cDNA clone image as a template and introduced into the pCS2 vector^53^. The myc-N terminal tagged version of *Trpc1* was obtained by in-frame introduction of the *Trpc1* ORF into the pCS2–6 myc vector^54^. The MO-resistant *Trpc1* construct, named *r-trpc1*, was generated by PCR to introduce an Afel site downstream of the start codon of *trpc1*, which respects amino acid sequence, and its in-frame cloning into the pCS2–6myc vector generated 12 mismatches, compared to the sequence recognized by the TRPC1-MO1. All these constructs were sequence-verified. pCS2-BMPRII-HA (full length) was kindly provided by Dr Reiko Satow^55^ and pCS2-BRII-ΔTD-HA (i.e., with the tail domain deletion of the BMPRII C terminus) was kindly provided by Dr Yoshiki Sasai^56^. All the constructs were linearized at the NotI site, and the capped mRNAs were *in vitro* transcribed with the SP6 mMessage mMachine kit (Ambion).

### *RT-qPCR, In situ* hybridization (ISH) and immunohistochemistry

Total RNA from 20 animal caps was isolated using Qiagen RNeasy mini columns, and 400 ng were reverse transcribed using a QScript cDNA synthesis kit (Quanta BioSciences). Gene expression levels were quantified in triplicate by real-time PCR (EvaGreen, BioRad) using a BioRad CFX96 machine, relative to the house keeping gene *odc* (ornithine decarboxylase). The primer sets used here are listed in Supplementary Table S1.

To determine the isoform of *trpc1* expressed in the animal caps, a supplemental PCR was conducted with primers designed as described^26^ flanking the 21 bp-longer sequence containing an EcoRV restriction site present only in the *trpc1* long form (Forward: 5′-tgcaccctgtgtacagccaag-3′ and Reverse: 5′-tggatcctcctcagtcagcata-3′). PCR amplicons were digested by EcoRV and the digestion products analysed on polyacrylamide gel.

Whole-mount ISH was carried out according to Ma *et al*.^57^ in order to reduce background staining and improve visualization of low abundant mRNA. Antisense RNA digoxigenin-labeled probes were synthesized by using cDNA templates encoding *zic3*^30^ and *sox2*^34^. For *trpc1*, anti-sense and sense digoxigenin-labeled probes were synthesized with SP6 and T7 RNA polymerases (Promega), respectively, and full-length cDNA *trpc1* was used as the template^53^. To analyse the spatial localization of *Ca*_*v*_*1.2* and *trpc1* expression, stained embryos were embedded in 3% low melting agarose for vibratome sectioning. In addition, the *zic3* ISH data were quantified using ImageJ (National Institutes of Health, USA), such that the areas of *zic3* expression were selected by adjusting the “color threshold” to purple and then using “free hand selections”. The ratio of the area of *zic3* expression in the uninjected and MO ± mRNA-injected sides of each embryo was calculated. Immunohistochemistry analysis of *trpc1* expression was performed on approximately 70 µm-thick vibratome sections of embryos fixed at blastula and gastrula (stage 9 and stage 10.5). Trpc1 localization was revealed with the rabbit anti-Trpc1 polyclonal primary antibody (NB100–91315, Novus Biologicals; at a 1/20 dilution), and an Alexa-488-conjugated anti-rabbit secondary antibody (A11008, Thermo Fisher Scientific). The nuclei were labelled with To-Pro3 (Thermo Fisher Scientific; at 1/1000 dilution).

### Immunoprecipitation and western blotting

For immunoprecipitation analyses, 12 animal caps from control and microinjected embryos were lysed for 20 min on ice in SOFT buffer (50 mM Tris pH 7.4, 100 mM NaCl, 5 mM EDTA, 0.05% NP40, 1% Triton X100, supplemented with phosphatase and protease inhibitors; Roche). The lysates were centrifuged at 13K for 20 min at 4 °C. Supernatant containing 10 µg total protein was used as the input. In addition, supernatant containing 120 µg protein was then used for immunoprecipitation with the anti-HA or anti-Myc antibodies (Sigma-Aldrich), after which it was pulled down with A/G protein beads (Sigma-Aldrich), and separated via SDS-PAGE. The presence of Myc-tagged Trpc1 and HA-tagged BmprII (both the full length and the C-terminal truncated form) proteins were revealed by immunoblotting with anti-HA and anti-Myc followed by visualization using an enhanced chemiluminescent kit (ECL, Amersham). Quantifications were performed with ChemiDoc Touch, using Image Lab software version 5.2.1 (Bio-Rad).

### Intracellular free Ca^2+^ measurements

All experiments were performed on animal caps isolated from MO-injected blastula embryos (stage 9). Intracellular free Ca^2+^ measurements were performed using the fluorescent, cell permeant Ca^2+^-indicator, Fluo4-AM (Invitrogen) and temporal data were then acquired using a CCD intensified camera (C2400–80, Hamamatsu Photonics, Japan), using methods described previously^6^.

### Membrane potential measurements

To measure changes in membrane potential, animal caps were incubated for 15 min with the potentiometric fluorescent dye, bis-(1,3-dibutylbarbituric acid) trimethine oxonol (DiBAC_4_(3); Molecular Probes), as described previously^16,58^. DiBac4(3) accumulates in the cytoplasm according to a Nernstian distribution. The fluorescence response results from potential-dependent partition of dye molecules between the cells and the extracellular medium. The absolute calibration of optical V_m_ signals is not straightforward since it is an indirect measurement. It is therefore important to establish a correlation between light intensity and membrane potential. Depolarization results in an influx of the dye and consequently an increase in fluorescence. Conversely, hyperpolarization induces a decrease in fluorescence. The calibration is acheived by imposing a K^+^ diffusion with increasing external K^+^ concentration in the presence of 1 µM valinomycin^59–61^. The potential is given by the Nernst equation, Vm = −58 log ([K_i_]/[K_o_]), assuming that the K^+^ conductance in the presence of valinomycin is much larger than the remaining ionic conductance of the cell. DiBaC4(3) was used at a final concentration of 500 nM (from a stock solution of 500 µM, prepared in a 1:1 mixture of ethanol and DMSO, according to the manufacturer’s instructions). Temporal membrane data were recorded using a Nikon AZ-100 multizoom stereo microscope equipped with a Nikon C1 scanning head (Nikon, Japan), and the excitation and emission wavelengths used, were 490 nm and 510 nm, respectively.

To prevent the movement of animal cap cells during the intracellular Ca^2+^ or membrane potential measurements, no perfusion system was used for the addition of noggin into the extracellular medium. This meant that the delay between the addition of noggin and the onset of the biological response was variable but it still remained within a ~5 to 10 min time frame.

### Statistical analysis

In the RT-qPCR, intracellular free Ca^2+^ and membrane potential experiments, the error bars represent the standard error of the mean (SEM). Mann-Whitney test or one-way ANOVA with Bonferroni’s test were performed with GraphPad Prism5 software. *p < 0.05, **p < 0.01, ***p < 0.001 and ****p < 0.0001.

## Supplementary information

supplementary information
