# Supplementary material for: Trpc1 as the Missing Link Between the Bmp and Ca2+ Signalling Pathways During Neural Specification in Amphibians
Source: Sci Rep. 2019 Nov 5;9:16049. doi: 10.1038/s41598-019-52556-0 (PMC6831629; doi:10.1038/s41598-019-52556-0)
Supplement: Supplementary file 1 — supplementary information [file 41598_2019_52556_MOESM1_ESM.pdf]

TRPC1 AS THE MISSING LINK BETWEEN THE BMP AND Ca^{2+} SIGNALLING PATHWAYS DURING NEURAL SPECIFICATION IN AMPHIBIANS

Isabelle Néant[1], Ho Chi Leung[2], Sarah E. Webb[2], Andrew L. Miller[2], Marc Moreau[1] and Catherine Leclerc[1]*

SUPPLEMENTARY INFORMATION

Figure S1

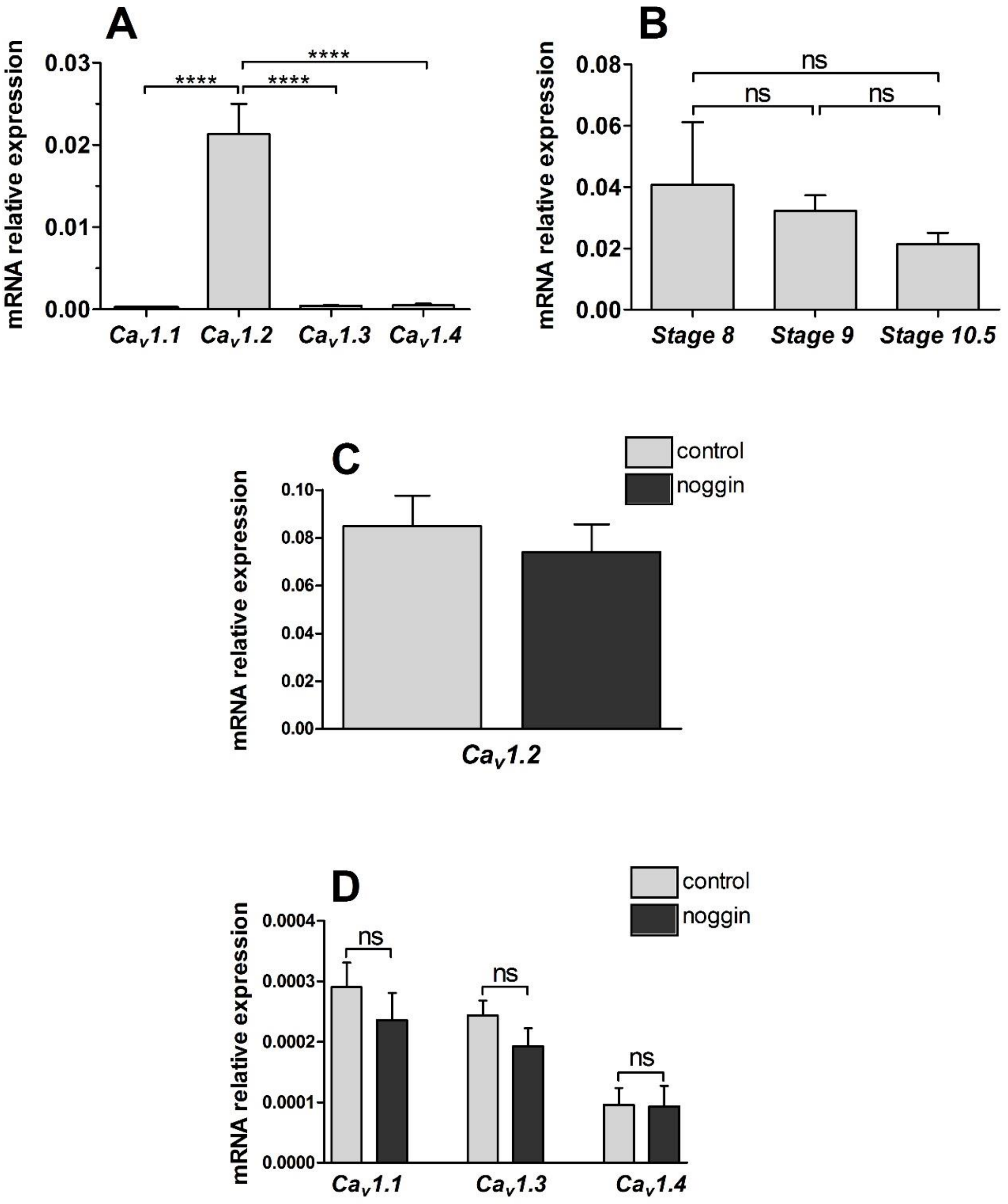

Figure S1: **Expression of *Ca*$_v$*1.x* mRNA in *X. laevis***. **(A)** RT-qPCR analysis of *Ca*$_v$*1.1, Ca*$_v$*1.2, Ca*$_v$*1.3* and *Ca*$_v$*1.4* in ectoderm (animal caps) isolated at mid-gastrula (stage 10.5). The expression levels were normalized to the housing keeping gene *odc (ornithine decarboxylase)*. The expression level *Ca*$_v$*1.2* mRNA was significantly higher than that of *Ca*$_v$*1.1*, *Ca*$_v$*1.3* and *Ca*$_v$*1.4*, in animal caps (one way ANOVA with Bonferroni's test, ****P<0.0001). The data represent the mean ± SEM of 4 independent experiments, with 20 animal caps being used for each experiment. **(B)** RT-qPCR analysis of *Ca*$_v$*1.2* in ectoderm (animal caps) isolated from embryos before gastrulation (stage 8 and stage 9) and at mid-gastrula (stage 10.5). The expression levels were normalized to the housing keeping gene *odc*

(ornithine decarboxylase). No significant changes in *Cav1.2* mRNA levels occurred during these 3 stages (one-way ANOVA with Bonferroni's test, $P<0.05$). The data represent the mean ± SEM of 4 and 5 independent experiments for stage 8 and 10.5, and for stage 9, respectively, such that in each experiment there were 20 animal caps. **(C-D)** Histograms to show RT-qPCR analysis of $Ca_v1.2$ **(C)** and of $Ca_v1.1$, $Ca_v1.3$ *and* $Ca_v1.4$ **(D)** in control stage 9 animal caps and in noggin-treated stage 9 animal caps. The level of expression was normalized to the housing keeping gene *odc (ornithine decarboxylase)*. No significant changes in the mRNA levels of $Ca_v1.1$, $Ca_v1.2$, $Ca_v1.3$, *and* $Ca_v1.4$ were observed when comparing control and noggin-treated animal caps (Mann-Whitney test). The data represent the mean ± SEM of 10 independent experiments, with 20 animal caps per experiment.

Figure S2

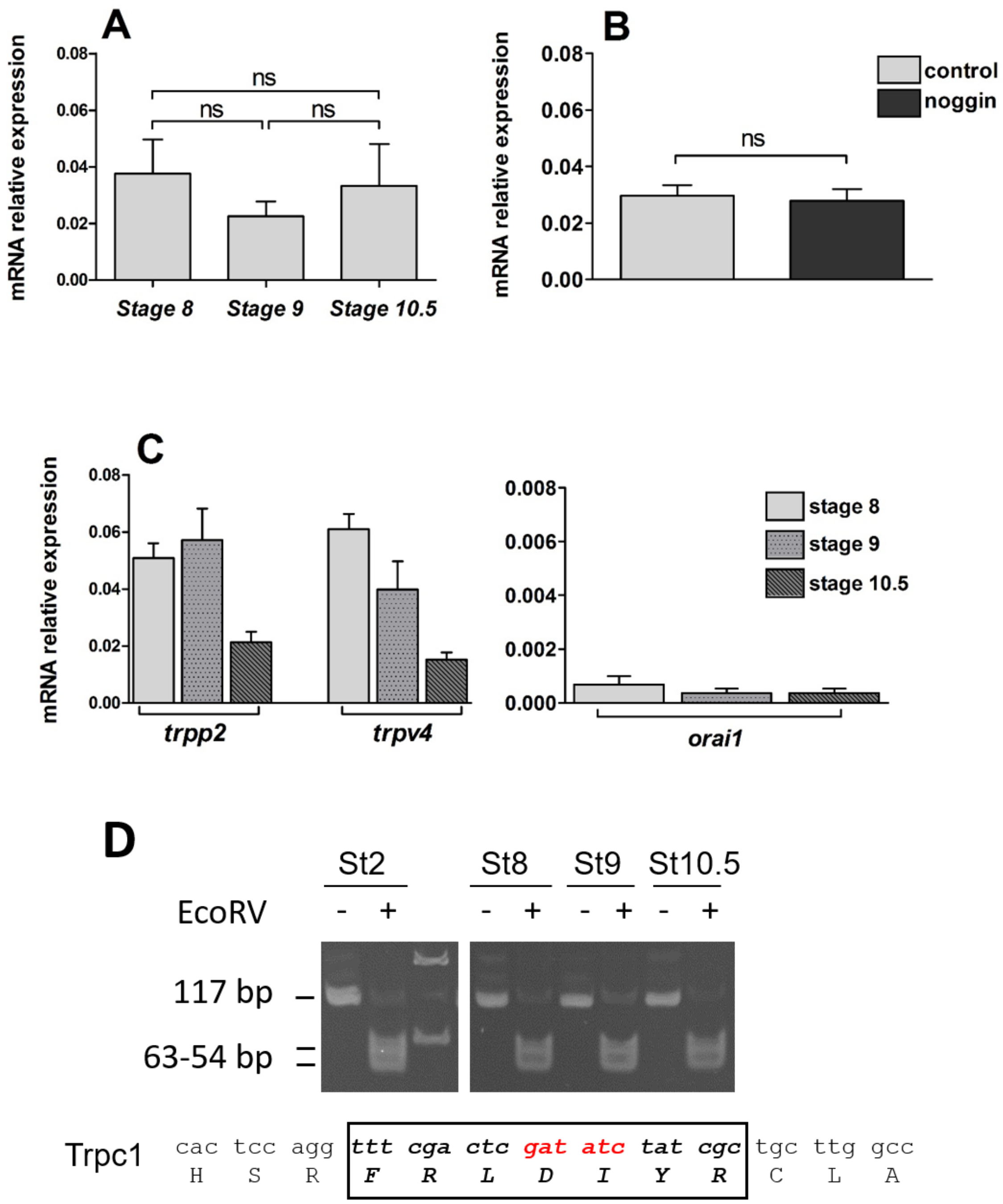

Figure S2: Expression of *trpc1, trpp2, trpv4* and *orai1* mRNA in *X. laevis*. (A) RT-qPCR analysis of *trpc1* in ectoderm (animal caps) isolated from embryos before gastrulation (stage 8 and stage 9) and at mid-gastrula (stage 10.5). In each case, the level of expression was normalized to the housing keeping gene *odc (ornithine decarboxylase)*. No significant changes in mRNA levels occurred during these 3 developmental stages (one-way ANOVA with Bonferroni's test, P<0.05). The data represent the mean ± SEM of 7 independent experiments for each of the 3 stages, and 20 animal caps were

collected for each experiment. **(B)** RT-qPCR analysis of *trpc1* in control stage 9 animal caps and in noggin-treated stage 9 animal caps. In each case, the level of expression was normalized to the housing keeping gene *odc (ornithine decarboxylase)*. When compared with the control animal caps, no significant changes in *trpc1* mRNA levels occurred in the noggin-treated animal caps (Mann-Whitney *t*-test). The data represent the mean ± SEM of 9 independent experiments, with 20 animal caps in each experiment. **(C)** RT-qPCR analysis of *trpp2, trpv4* and *orai1* in ectoderm (animal caps) isolated from embryos before gastrulation (stage 8 and stage 9) and at mid-gastrula (stage 10.5). In each case, the level of expression was normalized to the housing keeping gene *odc (ornithine decarboxylase)*. The data represent the mean ± SEM of 5 independent experiments for each of the 3 stages with 20 animal caps per experiment. **(D)** Early embryos and animal caps express the long *trpc1* isoform. EcoRV treatment of *trpc1*-PCR products systematically cleaved the 117-bp amplicon into 2 fragments of 63 bp and 54 bp as shown on the polyacrylamide gel. This was shown in maternal stage (Stage 2; St2) embryos as well as in animal caps at blastula (St 8 and St9) and gastrula (St 10.5). The sequence shows that the amplicon exhibits a conserved EcoRV site (in red), in the 21bp-longer isoform (bold in box). The full-length gels are presented in supplementary figure S8.

Figure S3

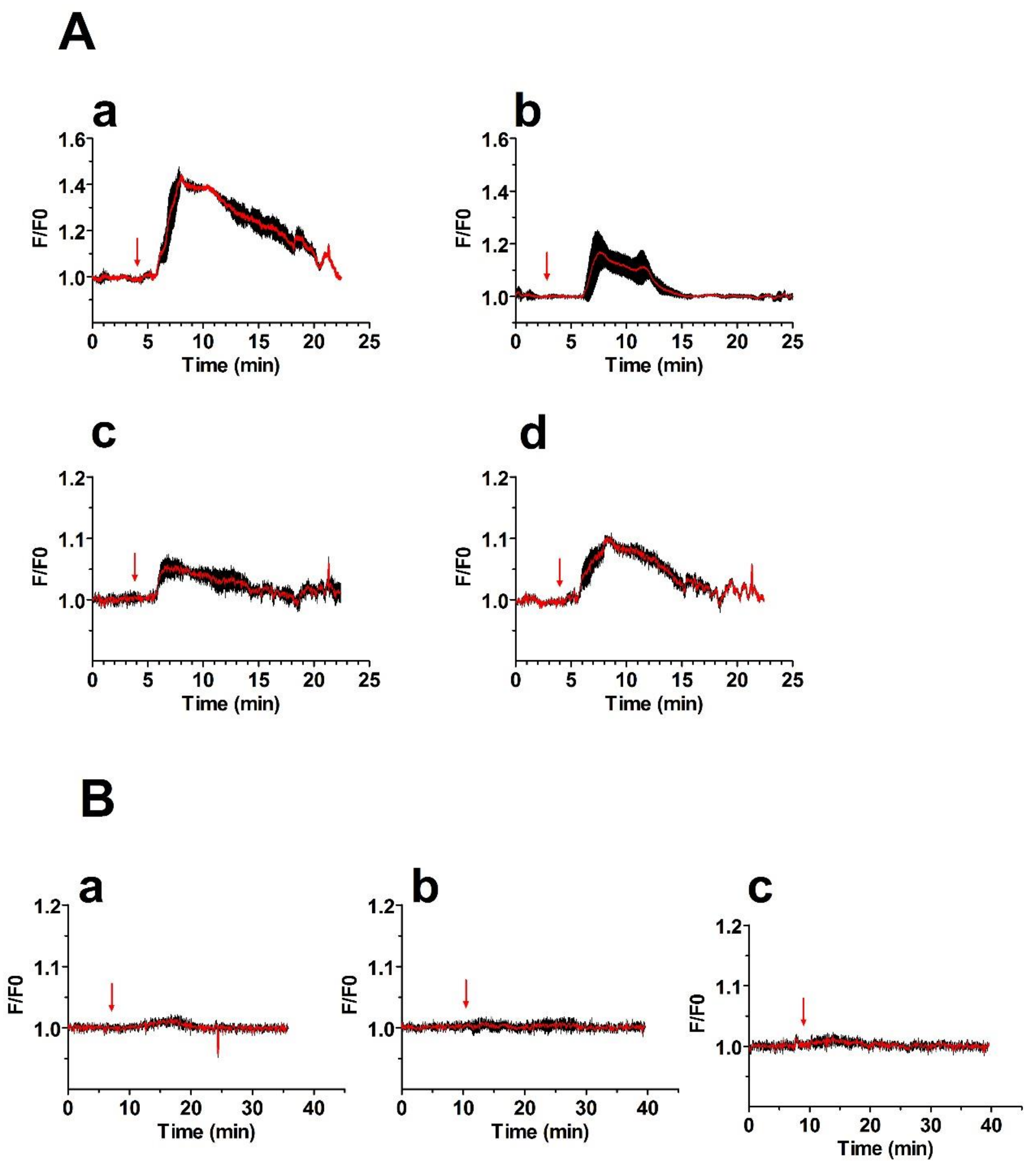

Figure S3: ***Trpc1* knock down abolishes the increase in intracellular Ca^{2+} generated following the activation of $Ca_V1.2$ channels in animal cap explants. (A-B)** Relative changes in fluorescence (F/F0) reveal changes in intracellular Ca^{2+} generated in a single animal cap loaded with the Ca^{2+}-indicator Fluo4 after being isolated from an embryo injected with either **(A)** control-MO or **(B)** TRPC1-MO1. In **(A)** panels **(a-d),** and in **(B)** panels **(a-c)**, the graphs show examples of 4 and 3 independent experiments, respectively. Values are plotted as the F/F0 mean (red traces) ± SEM (black bars) from 4 **(A)** or 10 **(B)** randomly selected fields within a single animal cap. Noggin (3 µg/mL) was added (blue arrows) within the first 10 min after the start of data acquisition.

Figure S4

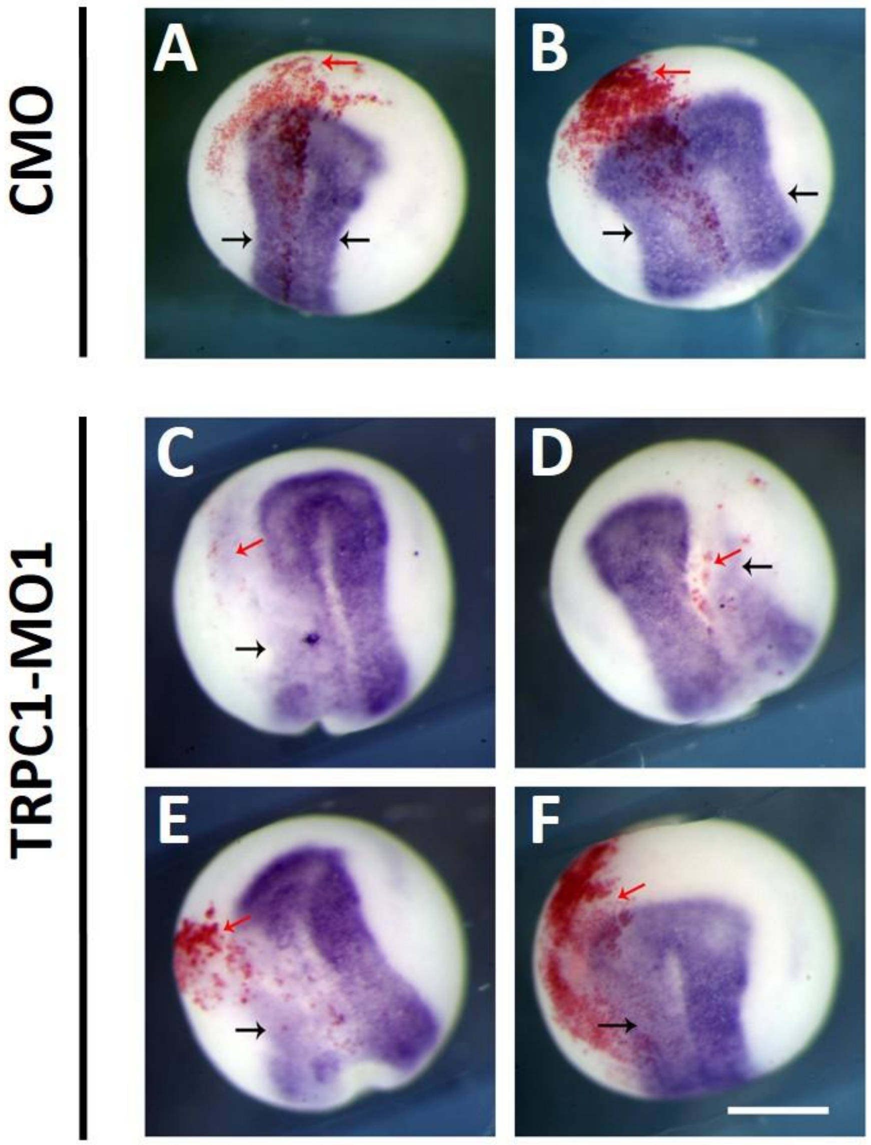

Figure S4: *trpc1* knock-down impairs the expression of the early neural gene, *sox2*. Embryos were co-injected at the 8-cell stage into a single dorsal animal blastomere with nuclear β-galactosidase mRNA (150 pg) and either **(A,B)** the standard control-MO (CMO; 17 ng) or **(C-F)** TRPC1-MO1 (17 ng). Embryos were then fixed at stage 14 for subsequent whole-mount *in situ* hybridization for *sox2;* see black arrows. The side of the embryo injected with MO was confirmed by reaction of β-galactosidase with Red-Gal, as shown by the red labelling on each embryo; see red arrows. **(A-B)** Images from 2 different embryos injected with CMO showing similar levels of *sox2* expression on the left and right side of the embryos. (**C-F)** Images from 4 different embryos injected with TRPC1-MO1 showing that the expression of *sox2* was reduced (see black arrows) on the injected side. Scale bar 500 µm.

Figure S5

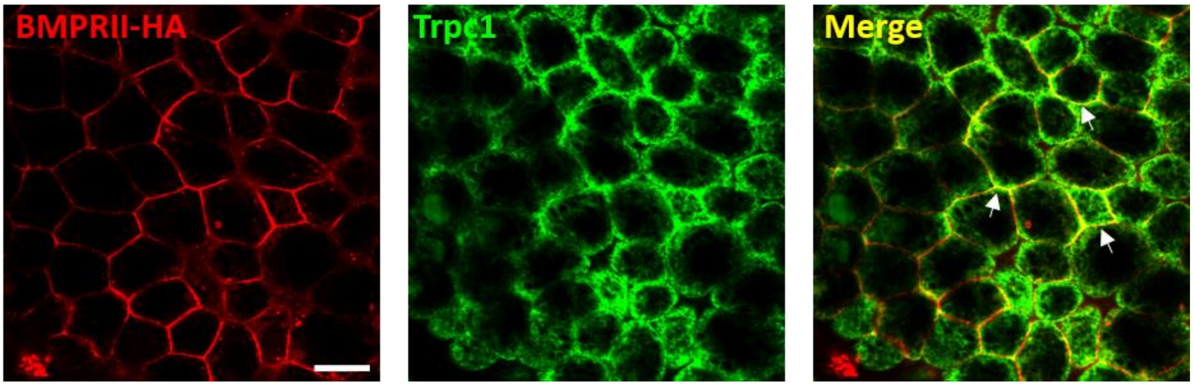

Figure S5: Trpc1 channels are co-expressed with BMPRII. Immunostaining of Trpc1 protein in anterior ectoderm of a representative stage 10 embryo. *Xenopus BMPRII-HA* (200 pg/cell) was injected into all the blastomeres of 4-cell embryos. The presence of HA-tagged BMPRII proteins and Trpc1 were revealed with an anti-HA antibody and a rabbit anti-Trpc1 polyclonal primary antibody, respectively. The secondary antibodies were Alexa-555-conjugated anti-mouse and Alexa-488-conjugated anti-rabbit for HA-tagged BMPRII and Trpc1, respectively. These images show the same confocal plane and indicate the co-expression of BMPRII-HA with Trpc1, which appears in yellow (see white arrows). Scale bar represents 30 µm.

Figure S6

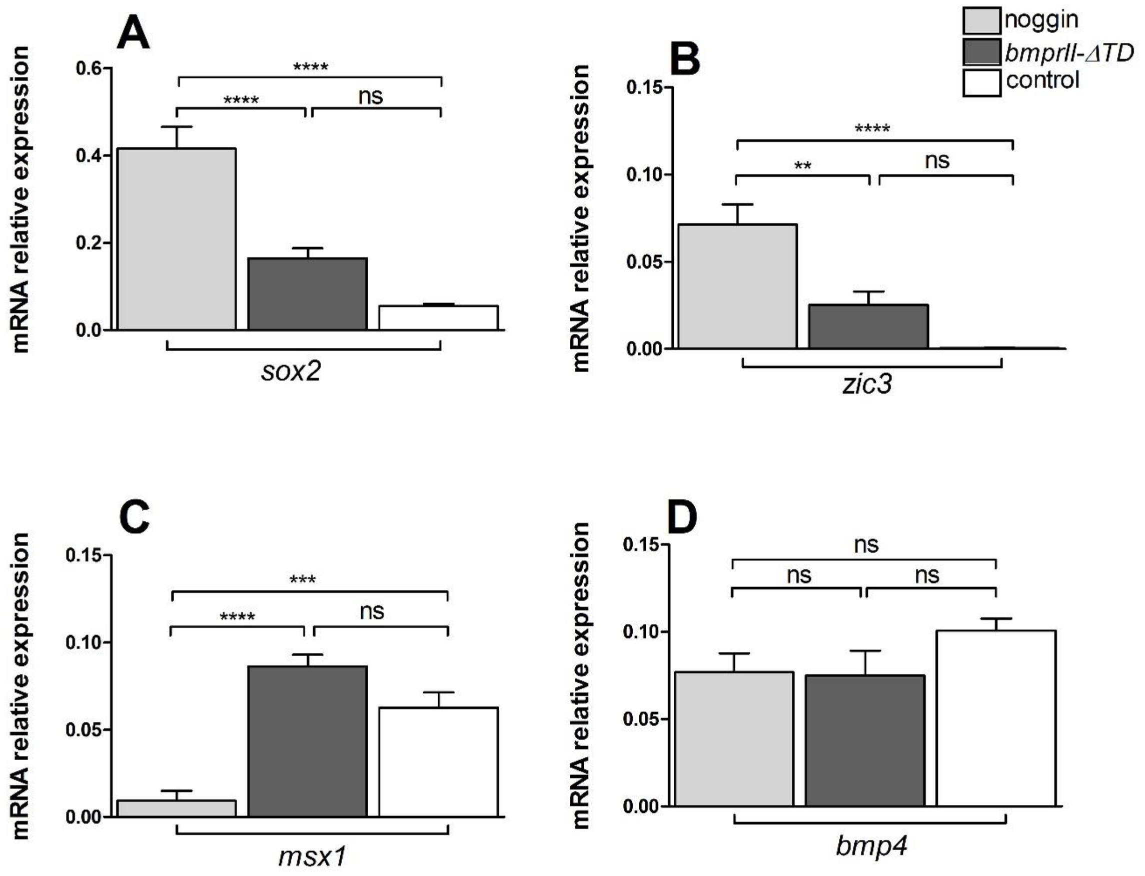

Figure S6. **The truncated form of BmprRII (BmprII-ΔTD) has no neural inducing activity**. RT-qPCR analysis of two neural genes, *zic3* **(A)** and *sox2* **(B),** and two genes controlling epidermal fate, *msx1* **(C)** and *bmp4* **(D),** in noggin-treated stage 9 animal caps (grey bars), in animal caps over-expressing *bmprII-ΔTD* (black bars) and in control animal caps (white bars). Expression was normalized to the housing keeping gene *odc (ornithine decarboxylase)*. When comparing the level of expression of these 4 genes in animal caps over-expressing *bmprII-ΔTD,* with the level of expression in noggin-treated and control animal caps, these data indicate that *bmprII-ΔTD* has no neural inducing activity, (one way ANOVA with Bonferroni's test, **P<0.001, ****P<0.0001). The data represent the mean ± SEM of 8 independent experiments, with 20 animal caps in each experiment.

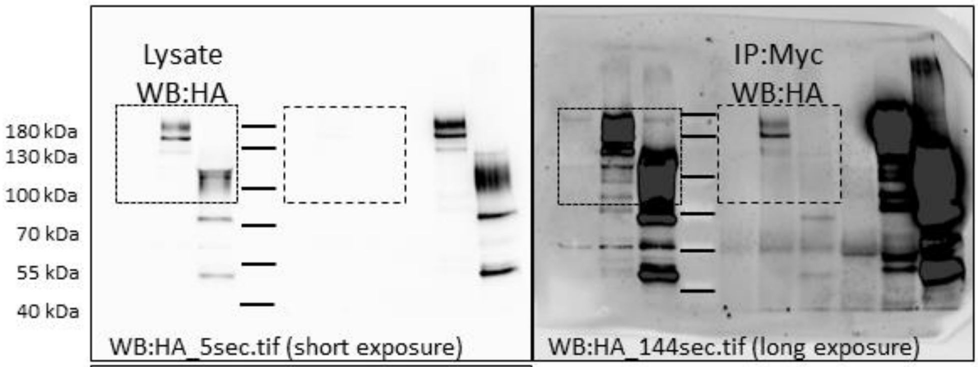

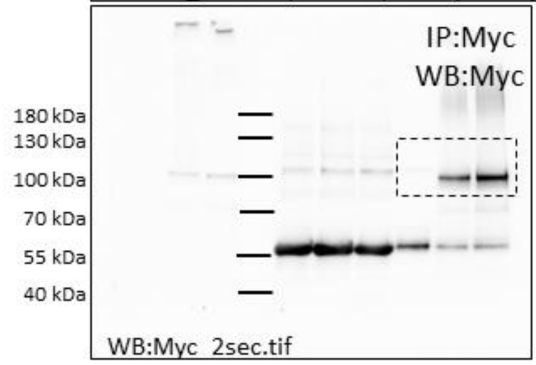

Full blot images of results shown in Figure 7B

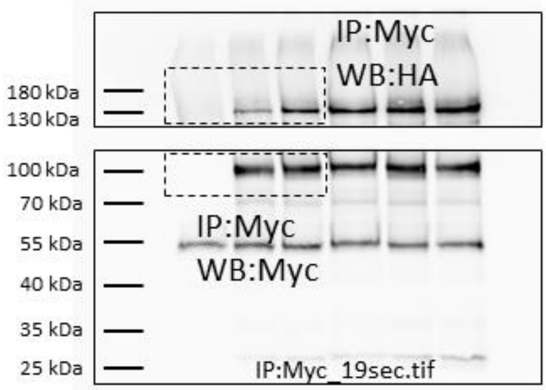

Full blot images of results shown in Figure 7C

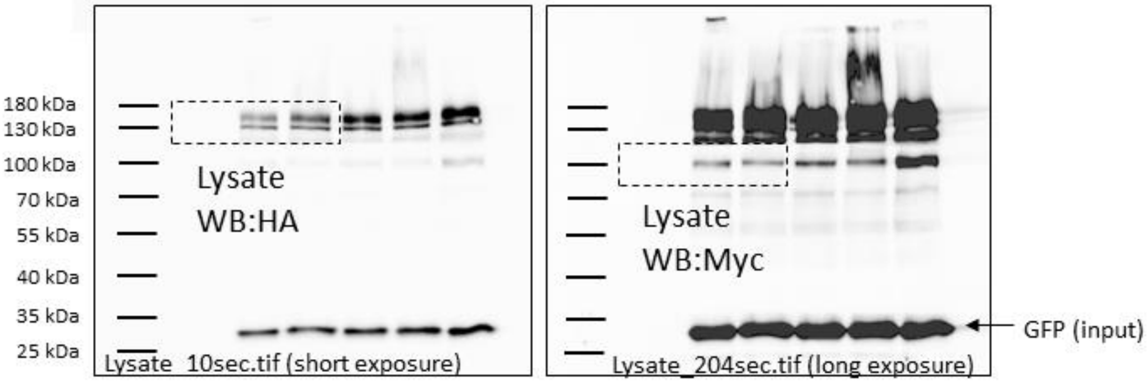

Supplementary figure S7 Full blot images of results shown in figure 7

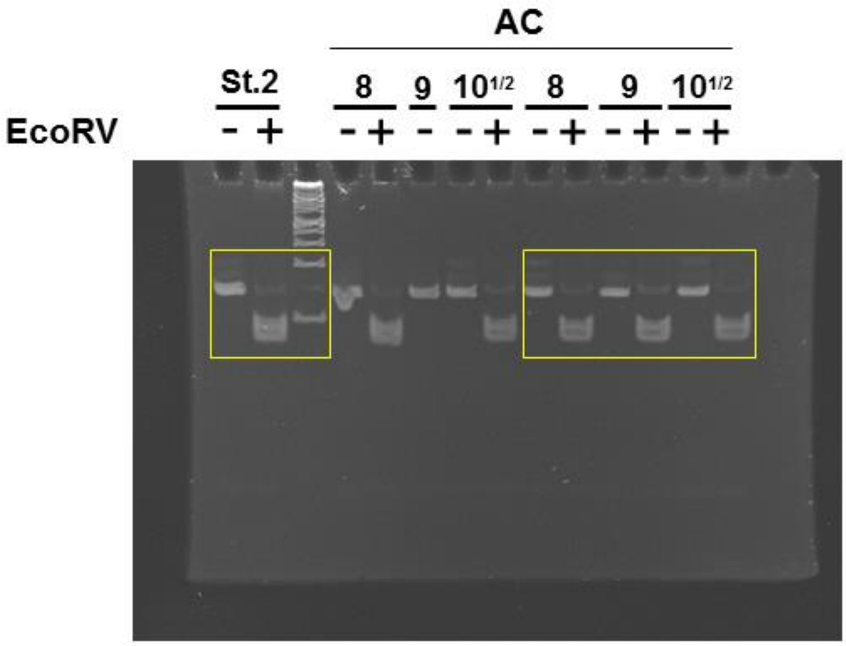

Supplementary figure S8: Full images of undigested and EcoRV-digested *trpc1*-amplicons on polyacrylamide gels shown in Figure S2D
